# Posttransplantation malignancy in a patient presenting with weight loss and changed bowel habits: a case report

**DOI:** 10.1186/1471-2369-7-9

**Published:** 2006-05-04

**Authors:** Roland Schmitt, Ute Kettritz, Friedrich C Luft, Ralph Kettritz

**Affiliations:** 1 Medical Faculty of the Charité, Franz Volhard Clinic, HELIOS Klinikum-Berlin, Germany

## Abstract

**Backround:**

Advancements in immunosuppressive therapy have significantly improved patient and graft survival following renal transplantation. This is paralleled by an increasing occurrence of posttransplantation malignancy.

**Case presentation:**

We report on a patient who presented with a history reminding of colon cancer seven years after receiving a kidney transplant. Initial diagnostic imaging seemed to confirm this diagnosis showing a constricting colonic lesion. To our surprise, colonoscopy findings were unremarkable. Review of the imaging studies revealed that the tumor-like picture was caused by the renal graft impressing the intestine. The following search for malignancy in other locations resulted in the diagnosis of glioblastoma multiforme of which the patient died several weeks later.

**Conclusion:**

Follow-up of renal transplant patients must include screening tests directed at tumor detection. Imaging studies and other tests in this patient group should be interpreted by physicians who are familiar with transplant related peculiarities.

## Backround

Kidney transplantation is the treatment of choice for appropriate patients with chronic renal failure. With advancements in immunosuppressive therapy patient and graft survival have significantly improved over the last decades. This achievement of long-term organ acceptance, however, coincides with an increased occurrence of posttransplantation malignancy [1]. We will present a renal transplant patient who was referred to us because of suspected colon cancer.

## Case presentation

A 66 year old male patient who had received a renal transplant seven years earlier because of IgA nephropathy in his native kidneys was referred to us by his nephrologist. His graft had functioned well and his creatinine was stable at 130 μmol/l with an immunosuppressive regimen of cyclosporine, azathioprine, and prednisolone that he had received since his surgery. Over the past 6 months he reported a 6 kg weight loss and increasing nausea. He had also noted loose, darkish stools several times daily and significant night sweats but no fever. He denied having travelled outside of Germany in the past year. His nephrologists had noted small contracted native kidneys, an unremarkable graft and normal remaining abdominal structures in an ultrasound study. A gastroscopy, chest x-ray and urological evaluation were unremarkable. All tests conducted for infectious diseases were negative. The haemoglobin and hematocrit, liver enzymes, thyroid hormone levels and serum proteins were normal. His creatinine was unchanged and the C-reactive protein was 4 mg/dl. His nephrologists, who suspected an intestinal malignancy in this transplanted patient, referred him to our service for colonoscopy and further evaluation.

**Figure 1:**
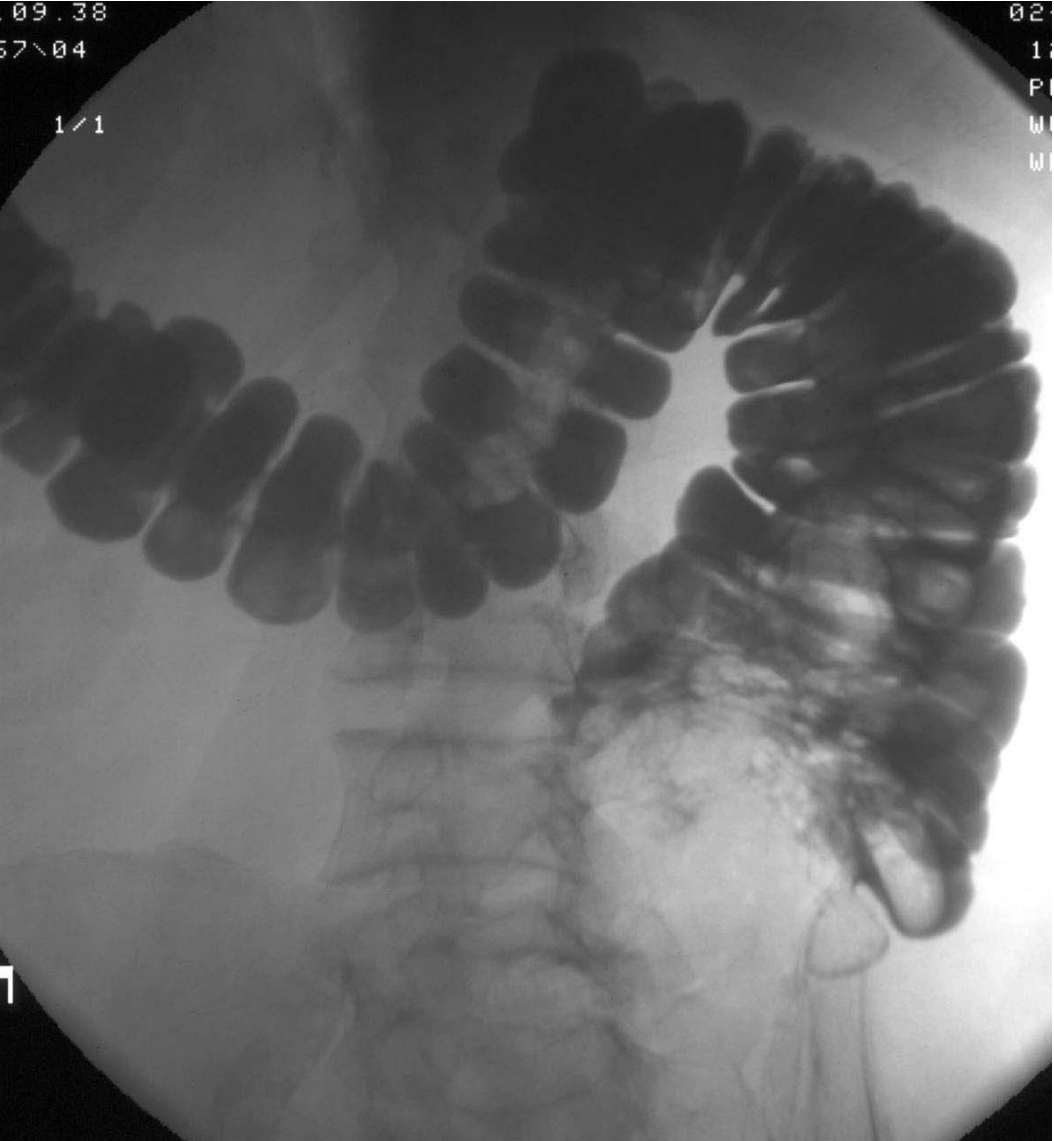
Water-soluble contrast study performed after instillation. The patient is lying prone. The cecum appears filled with a space-occupying lesion that the radiologist interpreted as highly suspicious of tumor.

**Figure 2:**
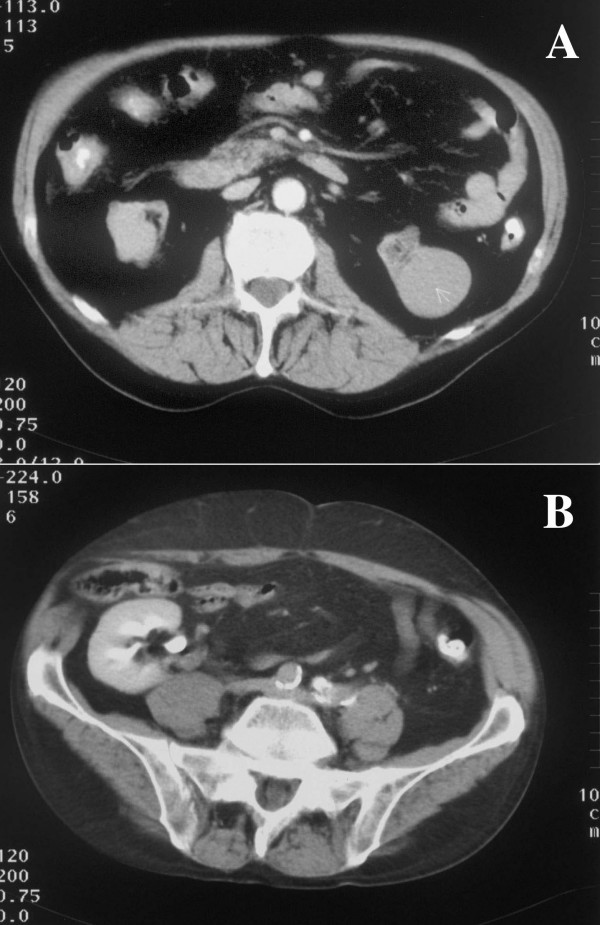
CT in the supine position. Panel A: The wall of the ascending colon was interpreted as 'thickened'. Panel B: CT at the level of the cecum showing the renal transplant kidney functioning well. The kidney is adjacent to the ascending colon and cecum.

**Figure 3:**
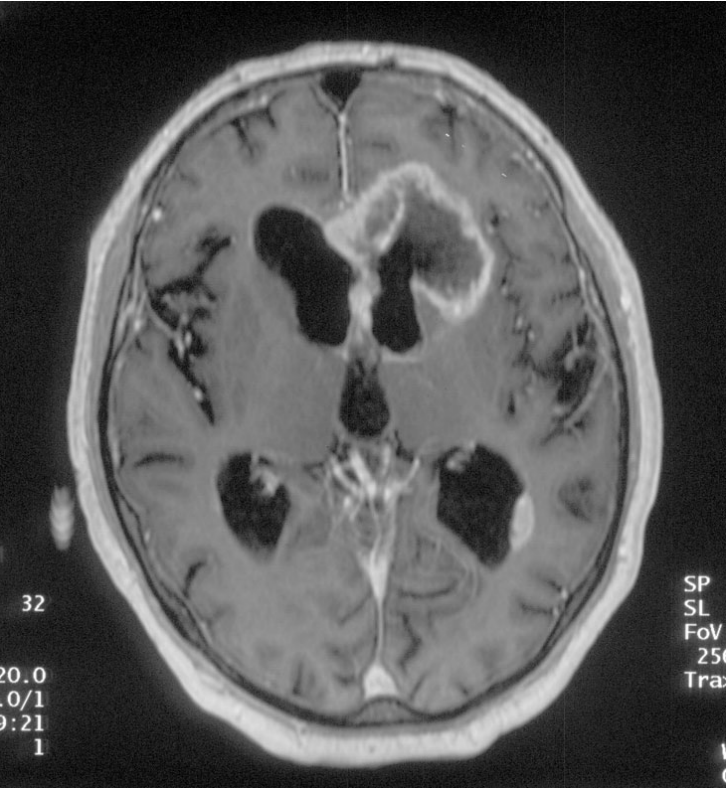
Magnetic resonance imaging of the brain revealed a left frontal, invasive, multilocular, infiltrating process.

The physical examination, including neurological examination was unremarkable. The blood pressure was 120/80 mmHg, the heart rate was 70/min. Our laboratory studies confirmed the ambulatory results. The cyclosporine level was in the therapeutic range. Serologic testing showed no signs of acute infection with adenovirus, Epstein-Barr Virus, Chlamydia pneumoniae, or Borrelia. PCR for CMV-DNA was negative. A colonoscopy was performed but was only successful to the level of the proximal sigmoid colon. The examination was terminated because of pain and definite resistance the gastroenterologist could not pass. Other than scattered diverticuli, the mucosa was normal. We next scheduled a radiocontrast to visualize the colon. In this study a constricting lesion was encountered in the cecum which the radiologist identified as a typical sign of colon cancer (figure 1). A computerized tomography was performed which showed a concentric wall thickening of the ascending colon about 3 cm above the cecum, which according to the radiologist, was consistent with the diagnosis of colon cancer (figure 2, panel a). All other abdominal organs were unremarkable otherwise or consistent with the earlier ultrasound examination. Before surgery another attempt at colonoscopy was scheduled. To our surprise, the second colonoscopy, performed with additional analgesia and sedation, revealed, a completely normal examination including the cecum. To address the obvious discrepancy between this result and the prior radiological findings we consulted another radiologist. She noticed that the radiocontrast study was performed with the patient lying prone (figure 1). Moreover, she pointed out the proximity of the ascending colon to the renal graft as shown in the computerized tomography, performed in the supine position (figure 2, panel b). She suggested that with the patient in this position, the graft could squash the colon in such a fashion that the instilled radiocontrast agent would produce a constricting tumor-like image. With these new insights we were forced to start from scratch and to look for a different diagnosis explaining the patient's symptoms. During his hospital stay we had observed that the patient, although unremarkable in the general neurological examination, appeared slow and sometimes inappropriate. In a mini-mental examination we found a significant reduction in cognitive function. The neurologist also found no localizing signs, but additional psychomotor testing verified rapidly progressive early dementia. A magnetic resonance imaging study of the brain was performed, revealing an infiltrating, destructive, multilocular lesion in the frontal lobe, (figure 3). The process was consistent with a malignant tumor and less suggestive of an infectious process. The patient was transferred to the neurosurgical section where a brain biopsy secured the diagnosis of glioblastoma multiforme. Radiation therapy was started but over the course of the next few days the patient's general status deteriorated rapidly and he became progressively more somnolent. Radiation had to be stopped and 6 weeks after initial admission the patient died.

## Conclusion

A significantly increased incidence of malignancy is well documented in renal transplant recipients if compared to the general population [1]. With improvements in immunosuppressive regimens that prolong patient and graft survival, neoplastic disease has developed into a frequent long-term complication in kidney recipients. One study found that 40 percent of renal graft recipients had cancer after 20 years of immunosuppression [2]. Many types of posttransplant cancers display a more malignant course than the same type of cancer in non-transplant patients. This aggressive behaviour of posttransplant malignancy contributes to the associated mortality causing 26 percent of deaths in patients surviving transplantation for at least 10 years [3].

One of the contributing factors to posttransplant cancer is the immunosuppressive therapy [1]. The association of immunosuppressive therapy and cancer development is also known from non-transplant patients but the precise role of immunosuppressive drugs in carcinogenesis is not fully understood. There is experimental evidence that the inhibition of immunological responses, especially after giving anti-T cell antibodies, increases cancer susceptibility by interfering with the host's anti-cancer surveillance system [1,4]. Accordingly, the amount of immunosuppression would be directly correlated to the risk of cancer. While this applies clinically to renal graft recipients on high-dose versus low-dose cyclosporine [5] it has also been demonstrated that cyclosporine is still carcinogenic in mice without an immune system [6]. This is partly ascribed to a TGF-beta dependent mechanism as cancer progression could be inhibited by TGF-beta blockade [6]. Similar effects have been reported for tacrolimus which also promoted cancer growth in immunodeficient mice [7]. A direct carcinogenic potential has recently been highlighted for azathioprine which increases the risk of skin cancer through higher UVA radiation sensitivity and subsequent DNA mutations [8]. In contrast to these cancer promoting side effects of calcineurin inhibitors and azathioprine, inhibitors of target-of-rapamycin (TOR), such as sirolimus and everolimus, seem to inhibit primary and metastatic tumor growth [9,10]. TOR inhibitor treatment was associated with reduced TGF-beta levels and with attenuated VEGF signalling [9,10]. A recent multivariate analysis including more than 30.000 kidney recipients showed a three times higher incidence rate of posttransplant malignancy in patients on calcineurin inhibitors as compared to patients on sirolimus or everolimus [11]. Another study followed 15 renal transplant patients with Kaposi's sarcoma while they were switched from cyclosporine to sirolimus [12]. Three months after the switch all Kaposi's sarcoma lesions had disappeared while no graft rejection occurred and the serum creatinine stayed stable [12]. These and other studies indicate that TOR inhibitors have an equivalent immunosuppressive potency but a reduced risk of malignancy.

Guided by our patient's history we agreed with the referring nephrologist and suspected post-transplant malignancy. Considering the negative results of outpatient gastroscopy, x-ray and ultrasound together with reported changes in bowel habits, we wrongly assumed the colon as the most likely tumor location. This assumption seemed to be confirmed by the misinterpreted radiological findings. While the incidence of rectal cancer is decreased in transplant patients, the risk of colon cancer is significantly increased [13]. A general odds ratio hierarchy of tumor risk in kidney transplanted patients shows in decreasing order: Non-melanoma skin cancer, thyroid and other endocrine tumors, oral cavity cancers, cervix and vaginal cancers, non-Hodgkin lymphoma, renal, ureteral, and bladder cancer, colorectal cancer, lung cancer, and brain tumors [14]. Our patient's final diagnosis was glioblastoma multiforme, which together with other brain tumors has an odds ratio of 2.5 in transplant patients. Although we were not able to help our patient this case illustrates several aspects:

1. Transplant patients need a regular history, physical examination and screening tests directed at tumor detection (reviewed in reference 1).

2. The prevalence and the behaviour of specific types of cancers in transplant recipients are significantly different from the general population.

3. Post-transplant malignancy can only in part be explained by immunosuppression itself.

4. Imaging studies in transplant patients should be interpreted by physicians familiar with transplant related peculiarities.

## Competing interests

The author(s) declare that they have no competing interests.

## Pre-publication history

The pre-publication history for this paper can be accessed here:


